# Whole-genome resequencing of native and imported dairy goat identifies genes associated with productivity and immunity

**DOI:** 10.3389/fvets.2024.1409282

**Published:** 2024-07-08

**Authors:** Jianqing Zhao, Yuanpan Mu, Ping Gong, Baolong Liu, Fuhong Zhang, Lu Zhu, Chenbo Shi, Xuefeng Lv, Jun Luo

**Affiliations:** ^1^Shaanxi Key Laboratory of Molecular Biology for Agriculture, College of Animal Science and Technology, Northwest A&F University, Xianyang, China; ^2^Institute of Animal Husbandry Quality Standards, Xinjiang Academy of Animal Sciences, Urumqi, China

**Keywords:** dairy goat, whole-genome sequence, genetic diversity, selective signal, production traits

## Abstract

Understanding the differences in genetic variation between local Chinese dairy goat breeds and imported breeds can help germplasm innovation and molecular breeding. However, the research is limited in this area. In this study, whole-genome resequencing data from 134 individuals of both local and imported dairy goat breeds were analyzed, and their differences in genomic genetic variation, genetic diversity, and population structure were subsequently identified. We also screened candidate genes associated with important traits of dairy goats such as milk production (*STK3*, *GHR*, *PRELID3B*), reproduction (*ATP5E*), growth and development (*CTSZ*, *GHR*), and immune function (*CTSZ*, *NELFCD*). Furthermore, we examined allele frequency distributions for the genes of interest and found significant differences between the two populations. This study provides valuable resources for the study of genetic diversity in dairy goats and lays the foundation for the selective breeding of dairy goats in the future.

## Introduction

Goats (*Capra hircus*) were among the first animal species domesticated, providing humans with essential food and living resources including meat, milk, and fur (1–4). As a specialized milk-producing breed, dairy goats are an important part of many countries’ livestock and dairy industries (5–7). China plays a prominent role in global dairy goat breeding and goat milk production, contributing significantly to the global milk supply (8).

The history of breeding dairy goats in China can be traced back to the early 20th century, with a primary focus on improving the Saanen dairy goat and its hybrid offspring. After nearly a century of breeding, the current dairy goat breeds in China mainly consist of Xinong Saanen, Guanzhong, and Laoshan dairy goats (9, 10). As people’s living standards have improved in China in past decades, the demand for dairy products with high quality has increased. To meet such requirements, the breeding of dairy goats has primarily focused on traits associated with high yield and quality (11, 12). Currently, to improve the excellent characteristics of dairy goat breeds in China, most farms focus on improving dairy goat breeds through selective breeding and crossing with imported breeds. The imported breeds in China mainly come from New Zealand and Australia, which comprise Saanen, Alpine, and Toggenburg dairy goats (13, 14). Among them, Saanen is the most commonly imported breed for milk production enhancement, while other breeds are primarily used to improve the milk quality of dairy goats (15, 16). However, the traits remain unclear, which hinders the verification of the breeding process. Meanwhile, dairy goat import primarily relies on phenotype evaluation without genomic level selection, which is not accurate nor very effective.

With the completion of the goat reference genome, significant progress has been made in the study of genetic diversity in dairy goats. These studies not only reveal genetic variations in the goat genome, but also provide important clues for understanding genetic differences and adaptation mechanisms among different breeds (17). However, previous studies have revealed the association between genetic variation and specific traits in the goat genome, research on the genetic variation and trait association of this specific population of dairy goats is still relatively limited (18–20). The existing research mainly focuses on a few major breeds, and the understanding of genetic differences between a wider range of dairy goat breeds is not yet in-depth. Therefore, in-depth exploration of the genetic diversity, population structure, and genetic variations associated with important agronomic traits of dairy goat populations is of great significance for the sustainable development of the dairy goat industry. In this study, we resequenced 50 local dairy goat genomes at 17.01× coverage depth. Then it was combined with the data from another 84 individuals of local and imported dairy goat species for further analysis to identify their differences in genomic genetic variation, genetic diversity, and population structure. Analysis of runs of homozygosity (ROH) islands and selective scanning identified specific regions and genes impacted by selection, associated with various significant morphological and agronomic traits. Additionally, we examined allele frequency distributions for the genes within selective signatures. Our findings contribute to the understanding of the genetic diversity and population genetic structure of dairy goats. Moreover, it revealed candidate genes that are associated with various phenotypes, which is valuable for future importation and breeding selection of dairy goats.

## Materials and methods

### Sample collection and sequencing

This study collected two populations of Xinong Saanen Dairy Goat (XNG, *n* = 40) and Guanzhong Dairy Goat (GZG, *n* = 10) from Shaanxi Province. Each goat had 5 mL of blood collected from the jugular vein, and DNA was extracted using the standard phenol-chloroform method. Samples with determined DNA concentrations were subjected to whole-genome sequencing by Huada Company, with paired-end libraries constructed using the Huada T7 platform having an average insert size of 500 bp per individual and an average read length of 150 bp. In addition, the resequencing data of 84 individuals were downloaded from public databases, including 8 breeds as follows: Australian Alpine Dairy Goat (AAG, *n* = 2), Australian Saanen Dairy Goat (ASG, *n* = 6), Guanzhong Dairy Goat (GZG, *n* = 26), New Zealand Saanen Dairy Goat (NSG, *n* = 6), New Zealand Alpine Dairy Goat (NAG, *n* = 4), Laoshan Dairy Goat (LSG, *n* = 9), Nubian Dairy Goat (NBY, *n* = 15), Tugenburg Dairy Goat (TGB, *n* = 16). Resulting in a total of 134 samples in the study.

### Read mapping and variant calling

The study utilized Trimmomatic v0.38 (21) for filtering the paired-end sequences. Next, BWA-MEM (v0.7.15-r1140) (22) aligned the clean data with the goat reference genome ARS1.2 (GCF_001704415.2, https://www.ncbi.nlm.nih.gov/datasets/genome/GCF_001704415.2/). Subsequently, samtools (23) was utilized to construct a BAM file index for mapping. The BAM files were then sorted and potential duplicate reads were removed utilizing the Picard^1^ tool. After mapping, SNP calling was performed using the “Haplotype Caller,” “Genotype GVCFs,” and “Select Variants” modules in the GATK genomic analysis tool package (GATK, version 3.8-1-0-gf15c1c3ef) (24). The initial SNPs were filtered with the “Variant Filtration” module based on the parameters: “QD < 2.0, FS > 60.0, MQ < 40.0, MQRankSum < −12.5, ReadPosRankSum < −8.0, and SOR > 3.0,” and an average sequencing depth of variants within the range “<1/3× and >3×” for all individuals. Lastly, the ANNOVAR (25) software was utilized for functional annotation of the SNPs.

### Genetic diversity analysis

After applying linkage disequilibrium (LD) to prune SNPs, the PLINK (26) program is utilized to compute runs of homozygosity (ROH) with specific parameters as follows: (a) 100 consecutive homozygous SNPs, (b) minimum of 50 SNPs per window, (c) 500 kb of contiguous homozygous length, (d) minimum density of 1 SNP per 50 kb, (e) window overlap rate of 0.05, and (f) each window containing 1 heterozygous and 2 missing calls. The subsequent analysis results are categorized into 0.5–1 Mb, 1–2 Mb, and 2–4 Mb for visualization. The heterozygosity of the SNPs is calculated to estimate the inbreeding coefficient (Fhom). VCFtools (27) are used to analyze nucleotide diversity for each breed in non-overlapping 50 kb windows. Next, the PopLDdecay (28) software is used to calculate the decay of LD to assess the physical distance between SNPs within haplotype blocks of different breeds. Finally, the frequencies of runs of homozygosity (ROH) islands for Xinong Saanen dairy goats and Guanzhong dairy goats are calculated, and regions with frequencies above 20% are determined as ROH hotspot islands.

### Population genomic analysis

Three methods are used for Variant Call Format (VCF) filtering and population structure estimation: (a) constructing a neighbor-joining tree with MEGA v10.2.6 (29) and visualizing it using iTOL (30); (b) principal component analysis (PCA) with EIGENSOFT v5.0 (31) software; and (c) conducting population structure analysis using ADMIXTURE v1.3.0 (32). Cross-validation is employed to calculate the cross-validation error and determine the optimal K value (assuming the ancestral population *K* value ranges from 2 to 8).

### Detection of selective sweeps

Based on the characteristics and origins of the dairy goat breeds, we categorized 134 dairy goats into native dairy goat (NDG) and imported dairy goat (IDG) breeds. We then compared the genomes of these two dairy goat breeds and estimated the signal scanning regions using a combination of nucleotide diversity (θπ NDG/IDG) and fixation index (FST) with a VCFtools, employing 50 kb sliding windows and 25 kb sliding steps. Additionally, Tajima’s *D* (33) statistic and cross-population composite likelihood ratio test (XP-CLR) (34) were employed to identify potential regional differences between different breeds. XP-CLR is a likelihood method for detecting selective sweeps by jointly modeling multi-locus allele frequency differences between two groups. The scanned regions detected from the intersection of the two parameters with the highest 5% threshold were annotated to identify candidate genes. Lastly, Bedtools (35) was used to annotate the selected regions for subsequent analysis.

### Enrichment analyses and visualization

The enrichment module in KOBAS (36) was utilized to identify pathways and Gene Ontology (GO) terms that showed statistically significant associations (at a significance level of *p* ≤ 0.05). Subsequently, the results were visualized through the OmicShare tool.^2^

## Results

### Sequencing and variation calling

**Figure 1:**
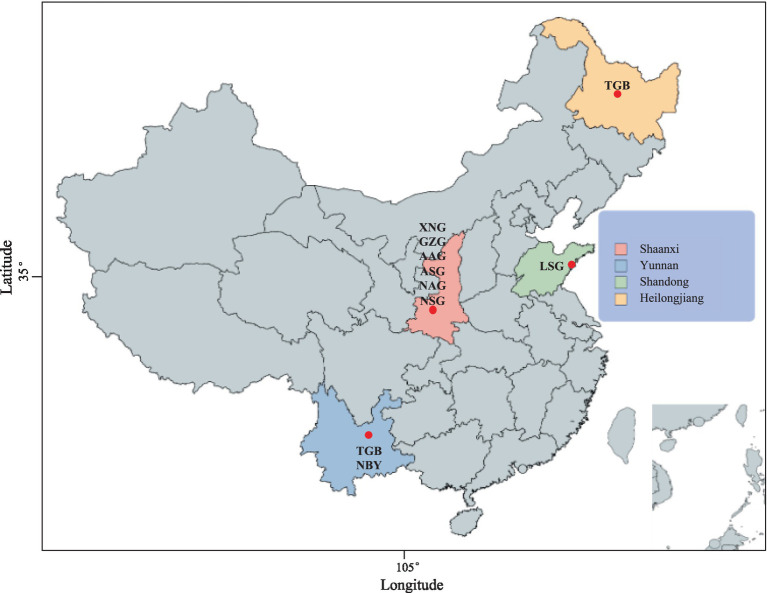
The geographic distribution of native dairy goat (NDG) and imported dairy goat (IDG) breeds. Xinong Saanen dairy goat (XNG, *n* = 40) and Guanzhong dairy goat (GZG, *n* = 10), Australian Alpine dairy goat (AAG, *n* = 2), Australian Saanen dairy goat (ASG, *n* = 6), Guanzhong dairy goat (GZG, *n* = 26), New Zealand Saanen dairy goat (NSG, *n* = 6), New Zealand Alpine dairy goat (NAG, *n* = 4), Laoshan dairy goat (LSG, *n* = 9), Nubian dairy goat (NBY, *n* = 15), Tugenburg dairy goat (TGB, *n* = 16).

To identify the genomic difference between local (XNG, GZG, LSG) and imported breeds of dairy goats (AAG, ASG, NSG, NAG, TGB, NBY), we compared 134 sets of whole-genome resequencing data, including 40 genomes of XNG and 10 genomes of GZG, combined with 84 published genome data that includes both local and imported breeds (GZG, LSG, AAG, ASG, NSG, NAG, TGB, NBY) (Figure 1). The data was aligned to the goat reference genome ARS1.2 (GCF_001704415), achieving an average alignment rate of 97.74% and a mean sequencing depth of 11.3×. After the removal of low-quality sequences, the average clean reads for each sample were 373,568,004 bp, with GC content ranging from 41.82 to 42.33%. The average alignment rate for the studied populations (XNG, GZG) is 97.5%, accompanied by an average sequencing depth of 17.01×. The imported breed populations (AAG, ASG, NSG, NAG, TGB) exhibited an average alignment rate of 97.04% and an average sequencing depth of 22.51× (Supplementary Table S1).

After SNP variation detection and statistical analysis of the obtained 134 dairy goat genome data sets, a total of 33,011,806 SNPs were detected. Among them, the proportion of intronic SNPs was the highest (32,705,301), accounting for 55.04% of the total; the proportion of exonic SNPs was approximately 1.142%. There were 283,685 synonymous SNPs and 227,235 missense SNPs. The transition/transversion ratio (Ti/Tv) was computed and analyzed, revealing a ratio of 2.344, consistent with the general value for goat populations. These findings indicate a high quality of sequencing, thus the obtained data meet the requirements for subsequent analysis (Supplementary Table S2).

### Genetic diversity analysis

**Figure 2:**
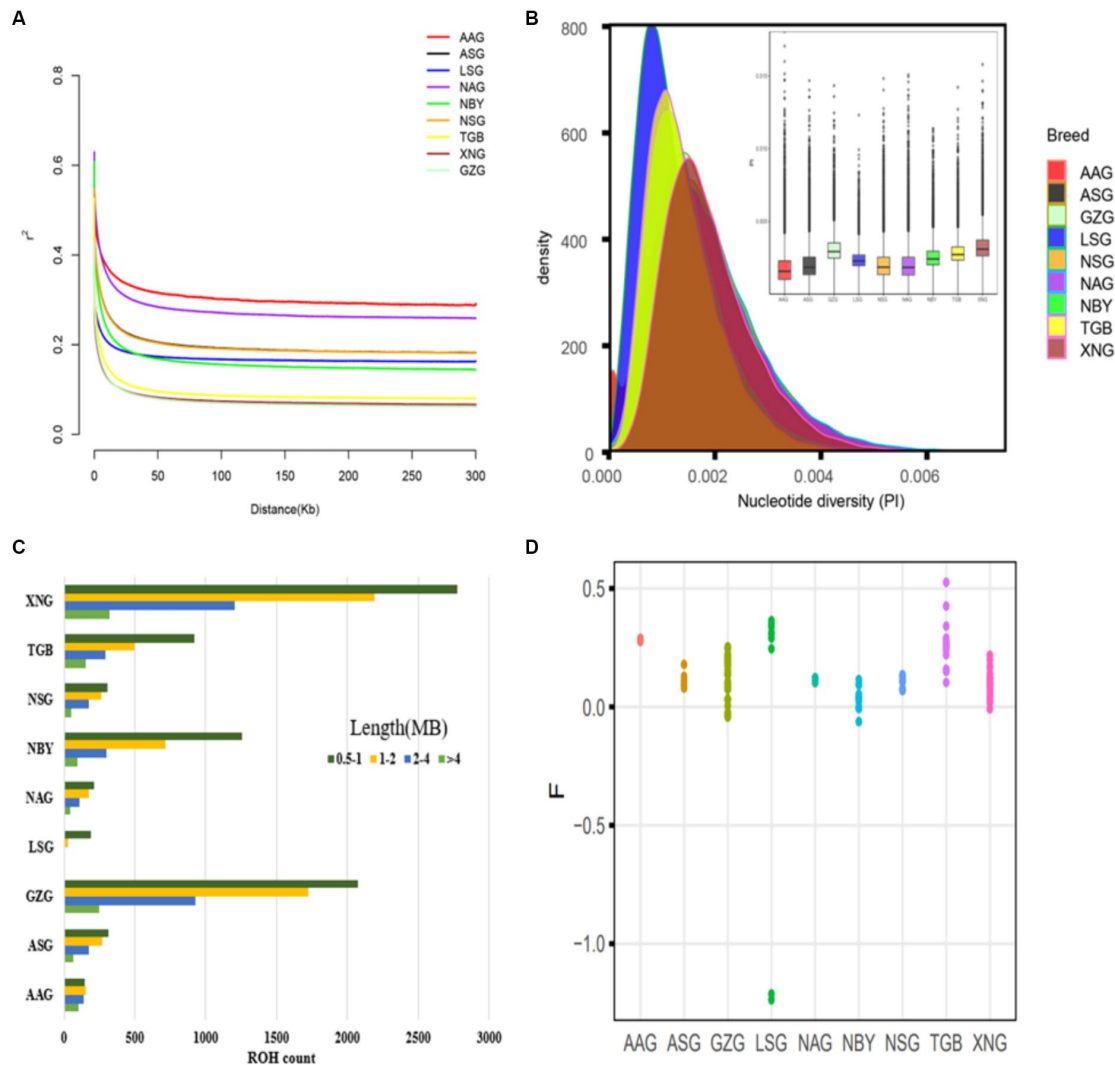
Genetic diversity among 134 samples from 10 populations. **(A)** Genome-wide average LD decay estimated from each group. **(B)** Density plots and Box plots of the nucleotide diversity for each group. **(C)** Estimation of the total number of ROH for each group. **(D)** Inbreeding coefficient for each individual.

The LD analysis shows that GZG and XNG exhibit the fastest decay rate (Figure 2A), suggesting lower domestication levels and higher genetic diversity. Nucleotide diversity analysis (Figure 2B) reveals higher diversity in local breeds compared to imported ones. ROH is an indicator of kinship proximity and inbreeding history, and the analysis reveals the genomic patterns of recent demographic history. Short ROH indicates that ancient inbreeding is much more prevalent in the XNG and GZG compared to other imported breeds. In addition, XNG exhibits a higher value of ROH, indicating a longer breeding history (Figure 2C). Meanwhile, the coefficient of inbreeding due to recent inbreeding is highest for each individual genome in TGB, with a range from 0.1 to 0.51 (Figure 2D).

**Figure 3:**
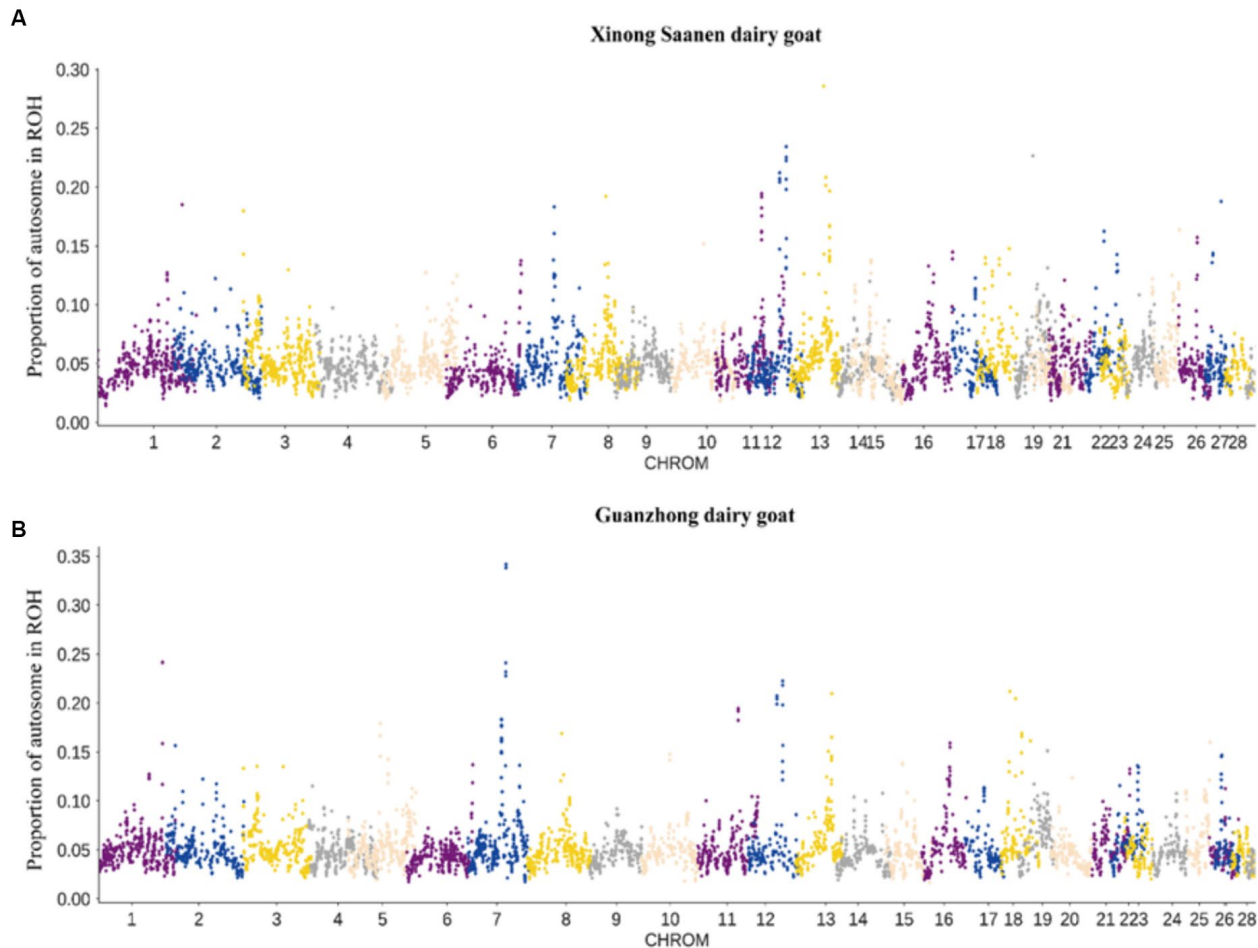
Manhattan plots of ROH frequencies. **(A)** Manhattan plots of ROH frequencies in Xinong Saanen dairy goat (XNG). **(B)** Manhattan plots of ROH frequencies in Guanzhong dairy goat (GZG).

This study conducted ROH island analysis on XNG and GZG. In the XNG samples, 16 ROH islands with a frequency of 20% were identified on chromosomes 12, 13, and 19 (Figure 3A), annotating to 31 genes. Similarly, on chromosomes 1, 7, 12, 13, and 18 of the GZG, 15 ROH islands with a frequency of 20% were identified (Figure 3B), annotating 25 genes. The Venn diagram (Supplementary Figure S1) illustrates 4 common genes between the two breeds (MPHOSPH8, LOC108637296, PSPC1, NBEA). In addition, the GO and KEGG enrichment analyses (Supplementary Figure S2) indicate enrichment of those identified genes in 23 biological processes (BP), 9 molecular functions (MF), and 2 cellular components (CC). The enriched pathways primarily include biological regulation, metabolic processes, immune system processes, and transcriptional regulation. Furthermore, the KEGG analysis reveals enrichment in valine, leucine, and isoleucine degradation, apoptosis, PI3K-Akt signaling pathway, and Rap1 signaling pathway.

### Phylogeny and population genetic structure

**Figure 4:**
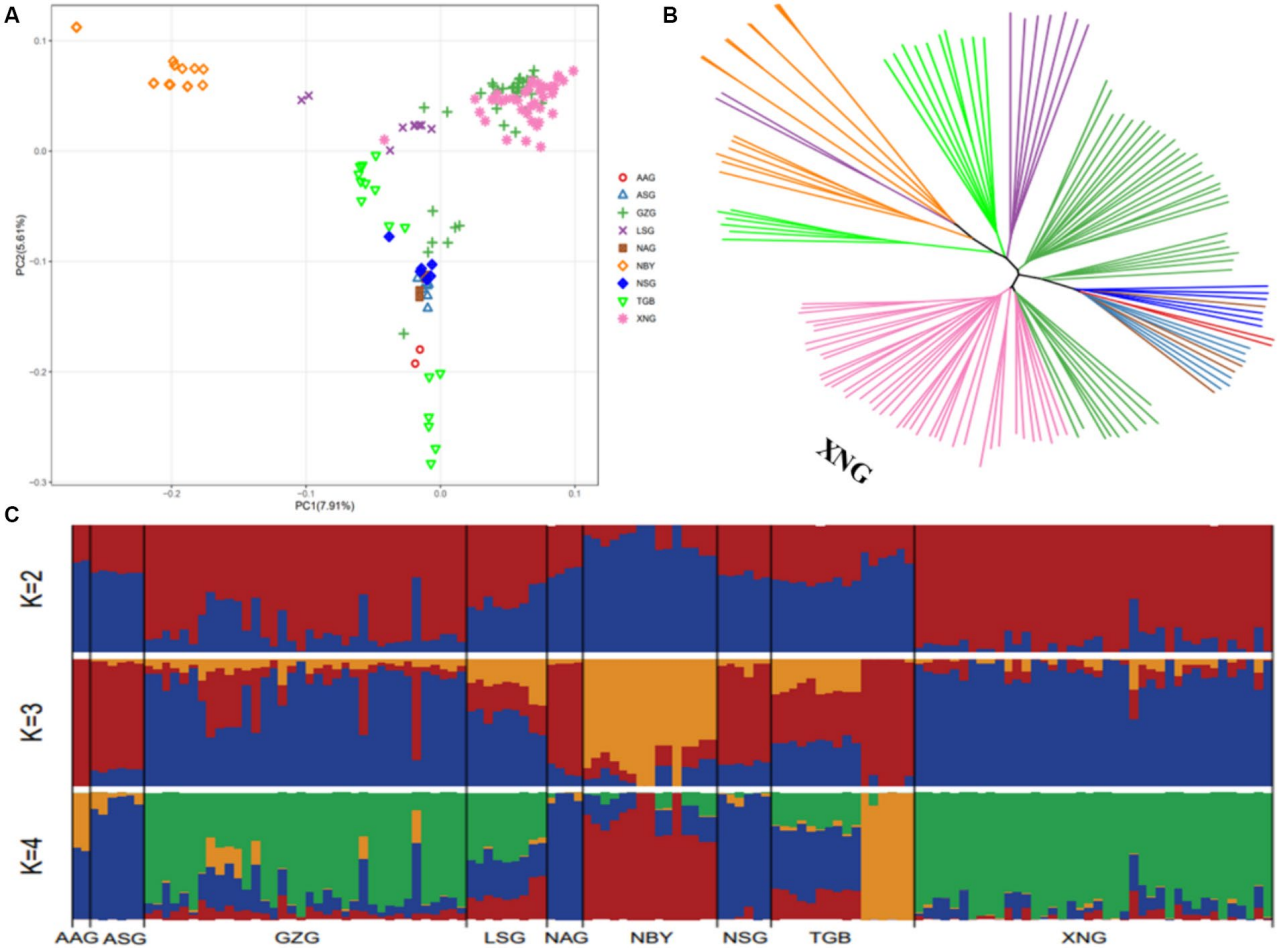
The structures of 134 samples from 10 populations. **(A)** PCA. Principal components 1 (7.91%) and 2 (5.61%) for the 134 dairy goats. **(B)** Phylogenetic tree. Phylogenetic relationships were estimated using the neighbor-joining method. **(C)** Genetic structure of cattle using ADMIXTURE when *K* ranged from 2 to 4.

To comprehend the population structure of local dairy goats in China, we looked into the SNP results derived from the resequencing data. Employing imported breeds as outgroups, we constructed SNP differentiation clustering based on various populations, the results of which can be used to mutually verify with other clustering methods. The percentages of PC1, PC2, and PC3 are 7.91, 5.61, and 3.60%, respectively, (Figure 4A; Supplementary Figure S3). There are three major clusters that AAG, ASG, LSG, NAG, NSG, and TGB are closely clustered, XNG and GZG form another cluster, while NBY is away from both of them. The result indicates that local breeds can be differentiated from imported breeds, and there are differences in clustering patterns among the 9 populations. The genetic data of the populations were utilized to calculate the degree of kinship between individuals, construct a genetic distance matrix, and build a phylogenetic tree utilizing the distance matrix. Although GZG and XNG are partially overlapped in the phylogenetic tree, the XNG group is clustered on its own, and imported breeds can be differentiated from local breeds, which is consistent with the PCA results (Figure 4B). In addition, for the 9 population, the number of subgroups (value of K) was preset to 2–9 for clustering, and the clustering results were cross-validated. The optimal number of subgroups was identified based on the minimum cross-validation error rate. *K* = 4 was identified as the optimal number of subgroups, and there were significant genetic differences among the XNG, NBY, NAG, and TGB populations. Regardless of the value of *K*, it consistently displayed genetic differentiation between local and imported breeds, indicating different genetic compositions among the populations, and relatively consistent genetic backgrounds among the different 9 populations (Figure 4C).

### Genome-wide selective sweep test

**Figure 5:**
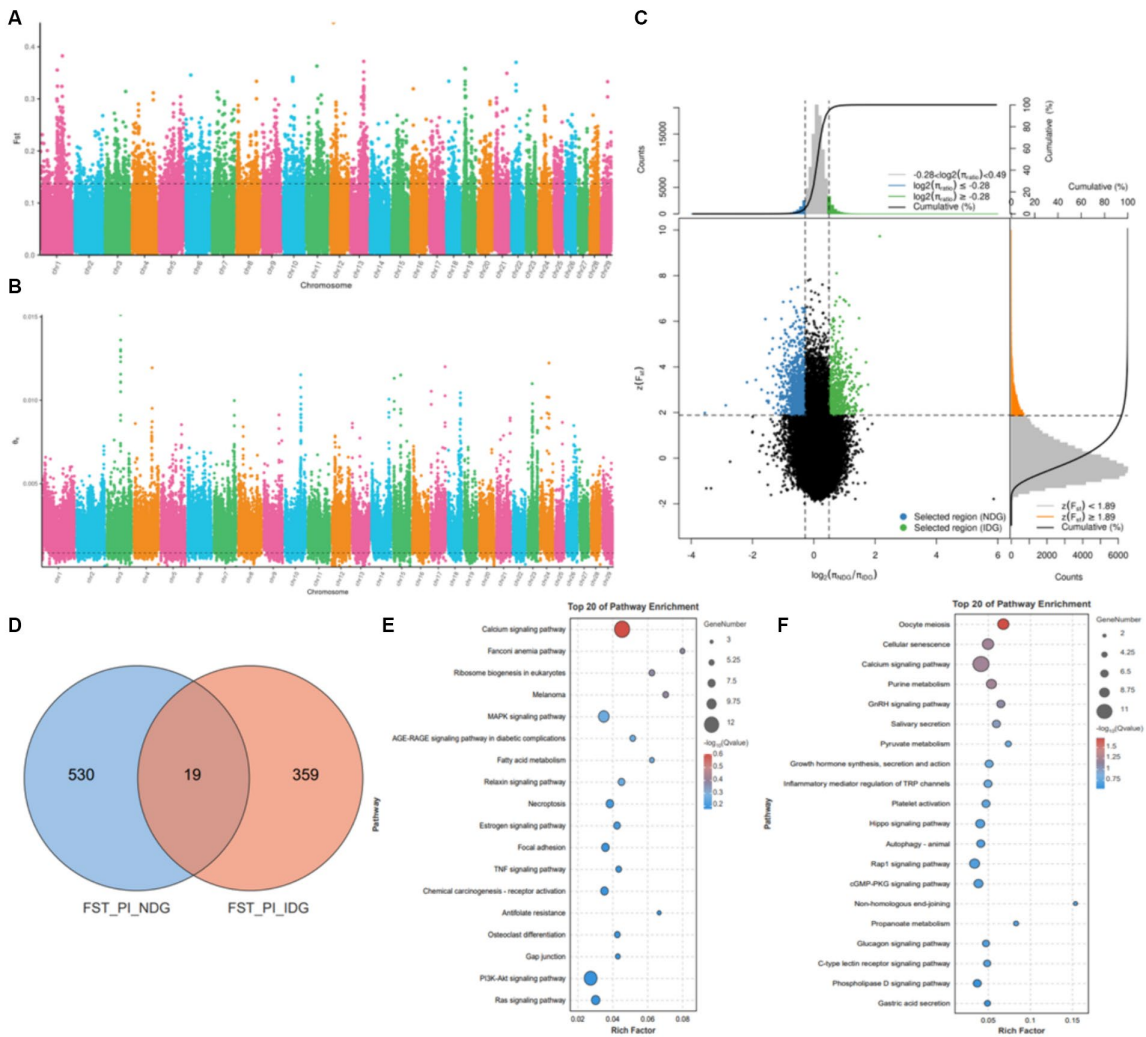
Selective sweep analysis of NDG and IDG. **(A)** Manhattan plot showing the FST. **(B)** Manhattan plot showing the θπ. **(C)** Distribution of log2 (θπ ratios) and FST values calculated in 50 kb sliding windows between NDG and IDG. **(D)** Venn diagram showing the gene overlaps among FST_PI_NDG and FST_PI_IDG. **(E)** KEGG pathway enrichment analysis based on genes across significant selective regions from NDG. **(F)** KEGG pathway enrichment analysis based on genes across significant selective regions from IDG.

To identify the regions of selection among the native dairy goat breeds and imported dairy goat breeds, we further conducted a comparative analysis using the resequencing data. Next, using the top 5% of both FST (Figure 5A) and θπ ratio (Figure 5B) cutoffs (Figure 5C), we observed that the native dairy goat breeds exhibited a total of 41.25 Mb across 607 selectively scanned regions. These regions encompassed 549 genes (Figure 5D) and showed significant enrichment in pathways such as the calcium signaling pathway, MAPK signaling pathway, fatty acid metabolism, and the PI3K-Akt signaling pathway (Figure 5E). On the other hand, imported dairy goat breeds displayed 25.05 Mb in 385 selective scanning regions, which encompassed 387 genes (Figure 5D). Pathway enrichment analysis revealed that those genes are involved in oocyte meiosis, cell senescence, calcium signaling pathway, GnRH signaling pathway, and the synthesis and secretion of growth hormone pathway (Figure 5F). These findings emphasize the genetic functional differences between local and imported breeds (Supplementary Figure S4).

### Selective signatures for traits

**Figure 6:**
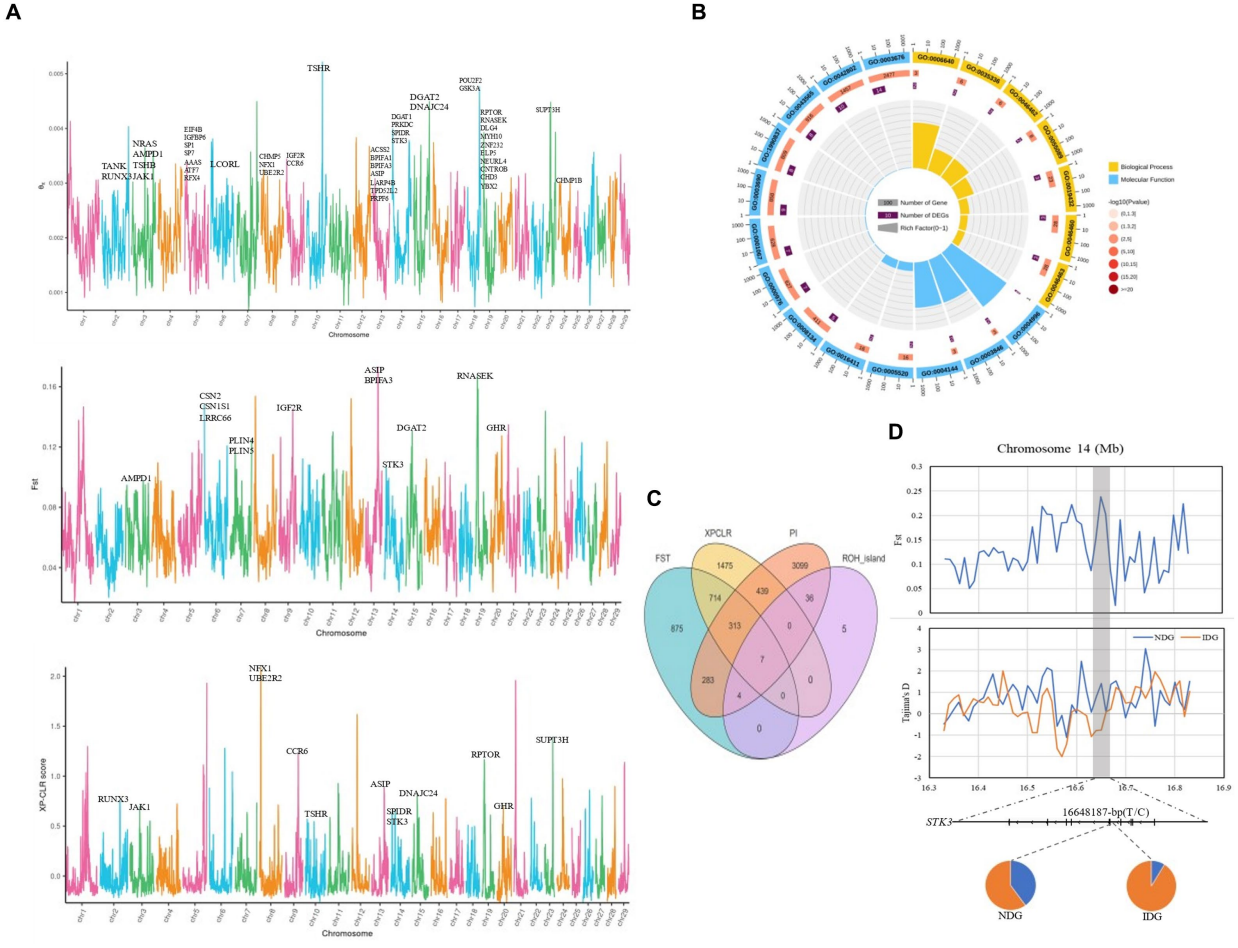
Genome-wide annotations during NDG and IDG. **(A)** Manhattan plot showing the θπ, FST and XP-CLR. **(B)** GO pathway enrichment analysis based on genes across significant selective regions. **(C)** Venn diagram showing the gene overlaps among FST, XP-CLR, PI and ROH_island. **(D)** FST value and Tajima’s *D* values around the *STK3* locus. Allele frequencies of SNPs within the *STK3* gene across the NDG and IDG, respectively.

To further explore the traits corresponding to selection signals, we performed FST, PI, and XP-CLR assays to discover positive selection in dairy goats. Meanwhile, for a more comprehensive annotation of genes We integrated and annotated the QTLdb database and a previously published genome-wide association analysis on important traits of dairy goats into our selection signaling results (Figure 6A). We found a large number of genes associated with milk production traits on chromosomes 5, 13, and 19 (Figure 6A), which were mainly involved in the monoacylglycerol biosynthetic process (GO:0006640), the long-chain fatty-acyl-CoA metabolic process (GO:00353336), monoacylglycerol metabolic process (GO:0046462), and fatty acid homeostasis (GO:0055089) (Figure 6B). In addition, these genes were also significantly enriched in the PI3K-Akt signaling pathway, glycerolipid metabolism, Fat digestion and absorption, and Regulation of lipolysis in adipocyte signaling pathways (Supplementary Figure S5). Integrative analysis (Fst, PI, XP-CLR) revealed two overlapping genes (Supplementary Figure S6), *ASIP* and *STK3*, respectively. Notably, integrative analysis (Fst, PI, XP-CLR, ROH_island) of the genes obtained from the above analytical methods revealed seven overlapping genes, including *PRELID3B*, *ATP5E*, *CTSZ*, *NELFCD*, *LOC108637417*, *TRNAF-GAA*, and *TRNAN-GUU* (Figure 6C). We then analyzed the allele frequency distribution of selected regions for *STK3*, *GHR*, and *PRELID3B* since many methods have detected these three genes, which showed that the gene frequencies of the selected genes differed between the local and imported breeds (Figure 6D; Supplementary Figure S7). Through the selection feature, we found that some regions were strongly selected. Some candidate genes were associated with milk production traits, growth and development, and immune traits.

## Discussion

Detecting recent positive selection signatures in domesticated animals that have gone through both artificial and natural selection can provide information on genomic sites that can contribute to the identification of beneficial mutations and underlying biological pathways for economically important traits. In this study, we analyzed the whole genome of 134 dairy goats from both native and imported breeds. In addition, the samples were obtained from populations that were also widely distributed in China, a feature that is absent in previous studies, which provides a more reliable background for the study. The detection of genetic variation, genetic diversity, and population structure showed that local dairy goat breeds are more genetically diverse than imported breeds. Previous studies in cattle (37, 38) and sheep (17) have shown similar results. This phenomenon suggests the local breeds have undergone less intensive selection, which may explain why their milk production performance is not outstanding. Therefore, crossing with imported breeds plus selection could be a promising way to improve the local breeds. However, detailed genetic information is needed to guide such a breeding process.

Milk quality traits are a complex biological issue that involves the interaction of multiple genes and biological pathways. Fatty acids, as one of the main components of milk fat, have a significant impact on the quality and characteristics of milk (39). By analyzing the selection signals of local and imported breeds for positive selection. we identified four genes that are involved in fatty acid metabolism (*HSD17B12*, *ACOX3*, *CPT1B*, and *CBR4*) in the selection signals of local dairy goats. The *HSD17B12* gene is involved in the synthesis of unsaturated fatty acids (40) and is also associated with reproductive performance (41). The *ACOX3* gene is associated with milk fat traits (42) and is a novel candidate for milk production traits (43). The *CPT1B* gene regulates long-chain saturated fatty acids and is involved in lipolysis (44, 45). The synthesis of fatty acids may involve *CBR4* (46). Notably, different pathway enrichments were observed in the imported breeds. *ACADS*, *AGPAT4*, *PTDSS2*, and *SMOX* are mainly involved in lipid metabolism and amino acid metabolism pathways. *ACADS* is involved in the fatty acid metabolism pathway (47), *AGPAT4* promotes triacylglycerol synthesis and fatty acid composition (48), *PTDSS2* is involved in the glycerophospholipid metabolism pathway (49), and *SMOX* is a candidate gene for growth, carcass composition traits, and milk production traits (50, 51). In addition, previously reported traits associated with immunity (*BPIFA3*, *LRRC66*) (52, 53), coat color (*ASIP*) (54), milk composition (*CSN1S1*, *DGAT2*, *PLIN4*, *PLIN5*, *IGF2R*) (55), and milk production (*AMPD1*) (56) were also found in imported breeds. Together, the results, suggest that the traits under selection were mainly associated with milk production, reproduction, and immunity for both the local and the imported breeds of dairy goats despite the difference in genes.

Milk production traits are the most important economic traits of dairy goats. This study includes milk production traits-associated gene markers from the QTLdb database for selection signal analysis. A large number of selected genes on chromosomes 13, and 19 were identified, which is similar to the results of a previous Genome-wide association study in dairy goats (15, 57). Through Fst, PI, and XP-CLR analysis, two genes with the highest selection signals showed up including *ASIP* and *STK3*. *ASIP* is responsible for regulating pigmentation and is associated with fat deposition and fatty acid composition in sheep (58, 59). The gene was identified as strongly selected due to the coat color difference between Alpine dairy goats (black) and native dairy goats (white). Whereas the *STK3* gene was identified as a new candidate gene for milk production traits (60), it also showed a strong selection signal in this study. We incorporated the results of the ROH_island analysis into the selective signaling analysis and identified seven genes that overlapped, including *PRELID3B*, *ATP5E*, *CTSZ*, *NELFCD*, *LOC108637417*, *TRNAF-GAA*, and *TRNAN-GUU*. *PRELID3B* has an important role in excessive melanin deposition (61) and regulates lipid accumulation in mitochondria (62). In lamb MII oocytes, down-regulation of *ATP5E* and up-regulation of *CUL1*, *MARCH7*, and *TRIM17* might cause low competence of lamb embryos (63). Furthermore, it has been reported that *ATP5E* is a promising candidate biomarker for oocyte viability after IVM (64), so the gene might be associated with reproductive traits in female animals. Many studies have demonstrated that *CTSZ* is linked to various traits, including immunity (65), pregnancy (66), regulation of thermogenesis in brown adipocytes (67), growth, carcass, and production (68). *NELFCD* may serve as a novel candidate gene for early immunomodulation (69) and it is strongly linked to *CTSZ* (70). *TRNAF-GAA* and *TRNAN-GUU* are tRNA-related genes, which might associated with immune (71) and reproductive traits (72, 73). The function of the gene *LOC108637417*, however, remains unclear. It is worth noting that the *GHR* is not only strongly selected, and also associated with various economic traits [milk production (74), growth (75), and milk quality traits (76)]. In summary, the identified genes in this study could be new candidate genes of milk production, reproduction, and immune traits in dairy goats.

To further investigate the differences of the genes associated with the selected regions in different breeds, the key genes *STK3*, *GHR*, and *PRELID3B* were subjected to allele frequency analysis. it was found that the genotypic frequencies of the strongly selected genes differed significantly among different populations. Similar results were found in chicken (77) and sheep (17) genomic research. By comparing the genetic differences between different varieties, we can better understand the genetic mechanisms behind these differences, providing guidance for future variety improvement and genetic resource protection.

## Conclusion

This study analyzed the genetic diversity, population structure, selection signals, and allele frequencies of local and imported dairy goats in China. It was found that there were significant genomic differences between the two populations. Moreover, candidate genes related to milk production (*STK3*, *GHR*, *PRELID3B*), reproduction (*ATP5E*), growth and development (*CTSZ*, *GHR*), and immune (*CTSZ*, *NELFCD*) traits were identified. It provides valuable insights into the genetic diversity of dairy goats and thus lays, the data support for future dairy goat breeding and selection in China.

## Data availability statement

The data that support the findings of this study are openly available in NCBI Sequence Read Archive at https://www.ncbi.nlm.nih.gov/, Accession number: PRJNA1127047.

## Ethics statement

The animal studies were approved by the Institutional Animal Care and Use Committee of Northwest A&F University. The studies were conducted in accordance with the local legislation and institutional requirements. Written informed consent was obtained from the owners for the participation of their animals in this study.

## Author contributions

JZ: Visualization, Writing – review & editing, Writing – original draft, Formal analysis. YM: Writing – review & editing, Data curation. PG: Writing – review & editing, Resources. BL: Writing – review & editing, Supervision. FZ: Software, Writing – review & editing, Methodology. LZ: Writing – review & editing, Methodology. CS: Writing – review & editing, Methodology. XL: Writing – review & editing, Resources. JL: Project administration, Writing – original draft, Writing – review & editing, Supervision, Conceptualization.

## Footnotes

^1^ http://broadinstitute.github.io/picard

^2^ http://www.omicshare.com/tools
