# Supplementary material for: Whole-genome resequencing of native and imported dairy goat identifies genes associated with productivity and immunity
Source: Front Vet Sci. 2024 Jul 8;11:1409282. doi: 10.3389/fvets.2024.1409282 (PMC11260678; doi:10.3389/fvets.2024.1409282)
Supplement: Supplementary file 1 [file Data_Sheet_1.pdf]

Supplementary Material

Whole-genome Resequencing of Native and Imported Dairy Goat Identifies Genes Associated with Productivity and Immunity

1. Supplementary Figures and Tables

1.1 Supplemental figures

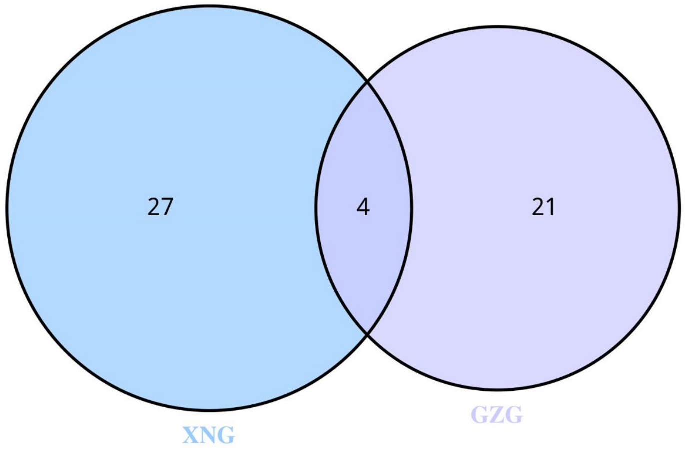

Figure S1 Venn diagram of genes identified in ROH islands analyses.

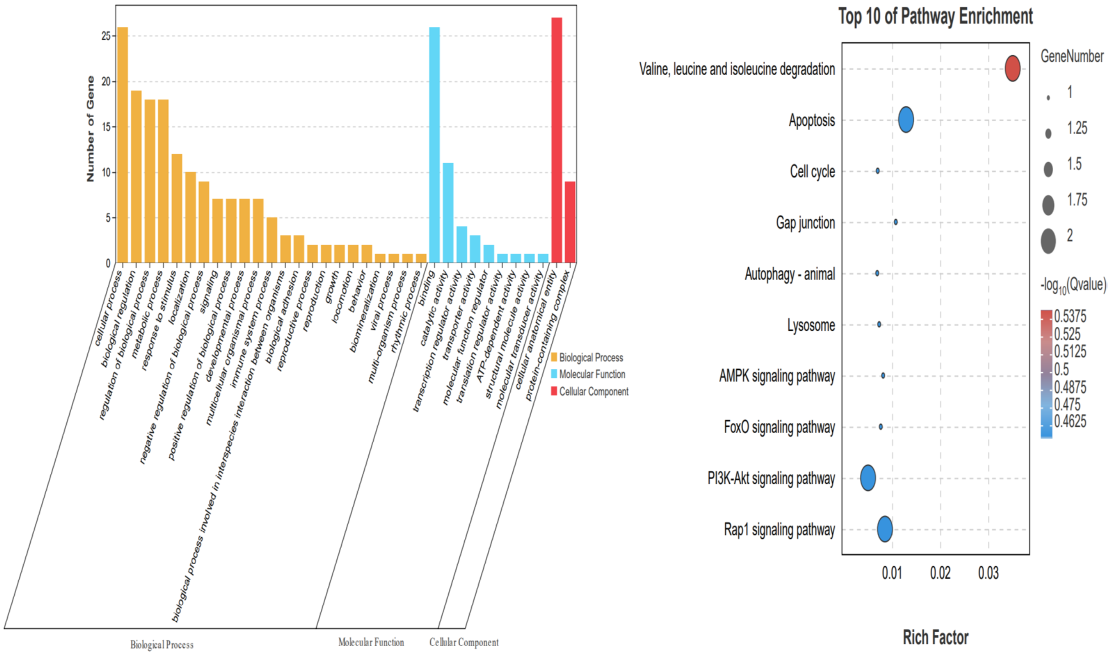

Figure S2 GO and KEGG analysis of genes identified in ROH islands analyses.

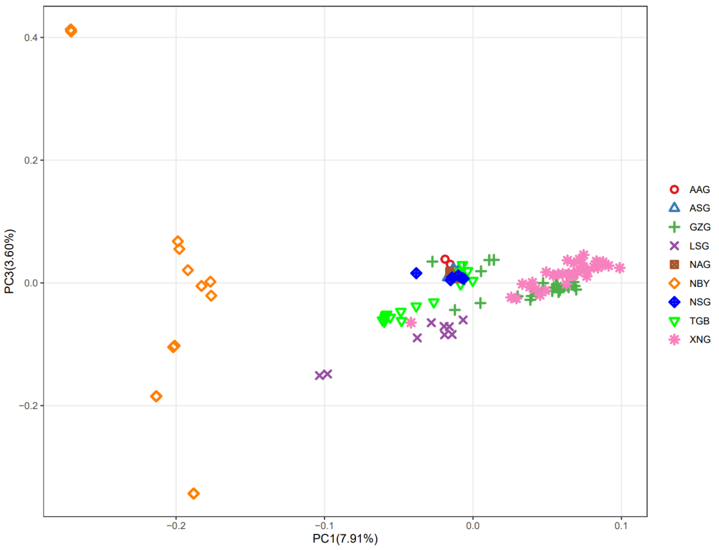

Figure S3 PCA. Principal components 1 (7.91%) and 3 (33.60%) for the 134 dairy goats.

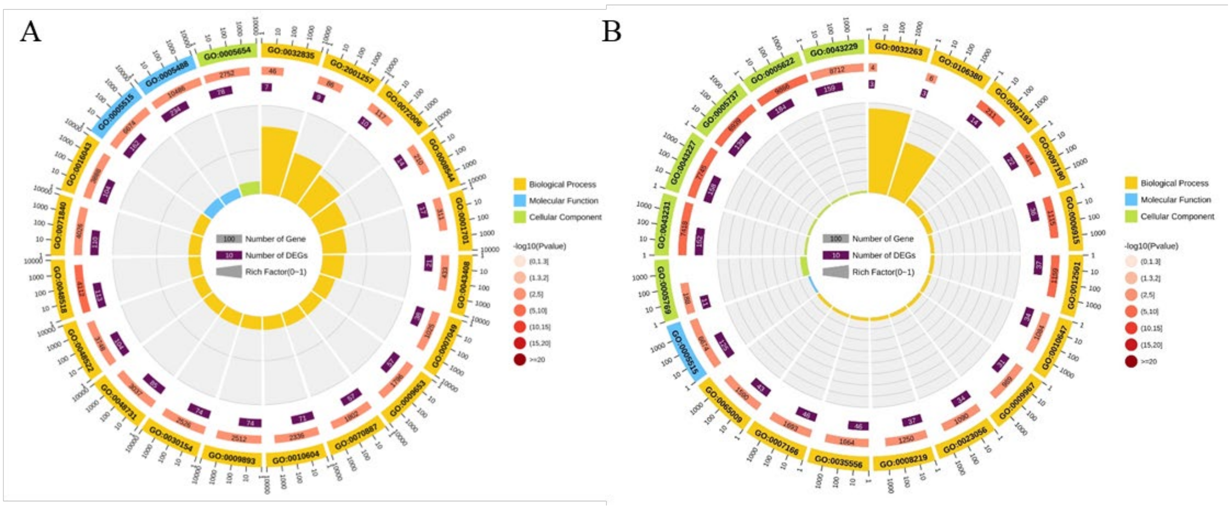

Figure S4 GO pathway enrichment analysis based on genes across significant selective regions. (A) NDG populations, (B) IDG populations

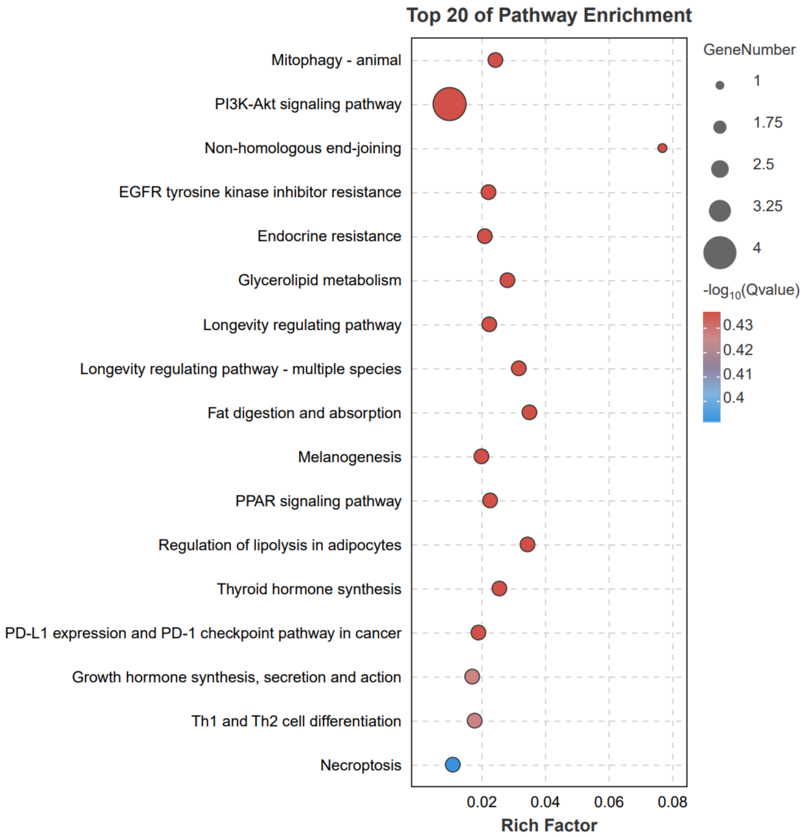

Figure S5 KEGG analysis of genes identified in selective signatures analyses.

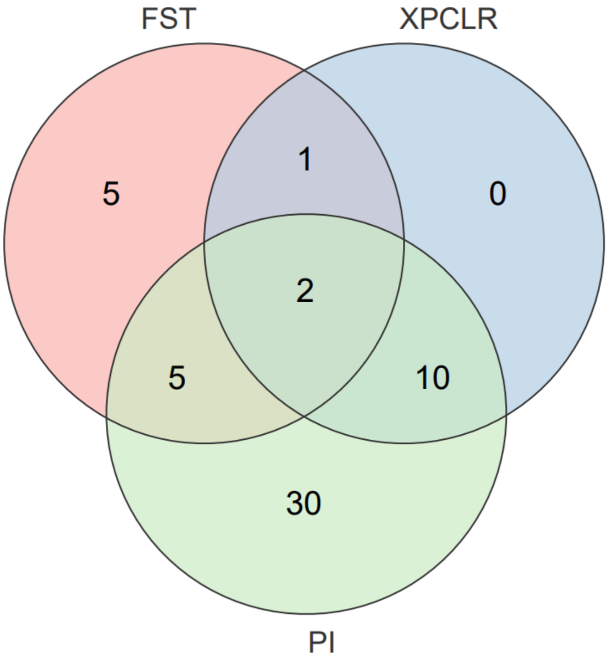

Figure S6 Venn diagram of genes identified in FST, XP-CLR, and PI analyses.

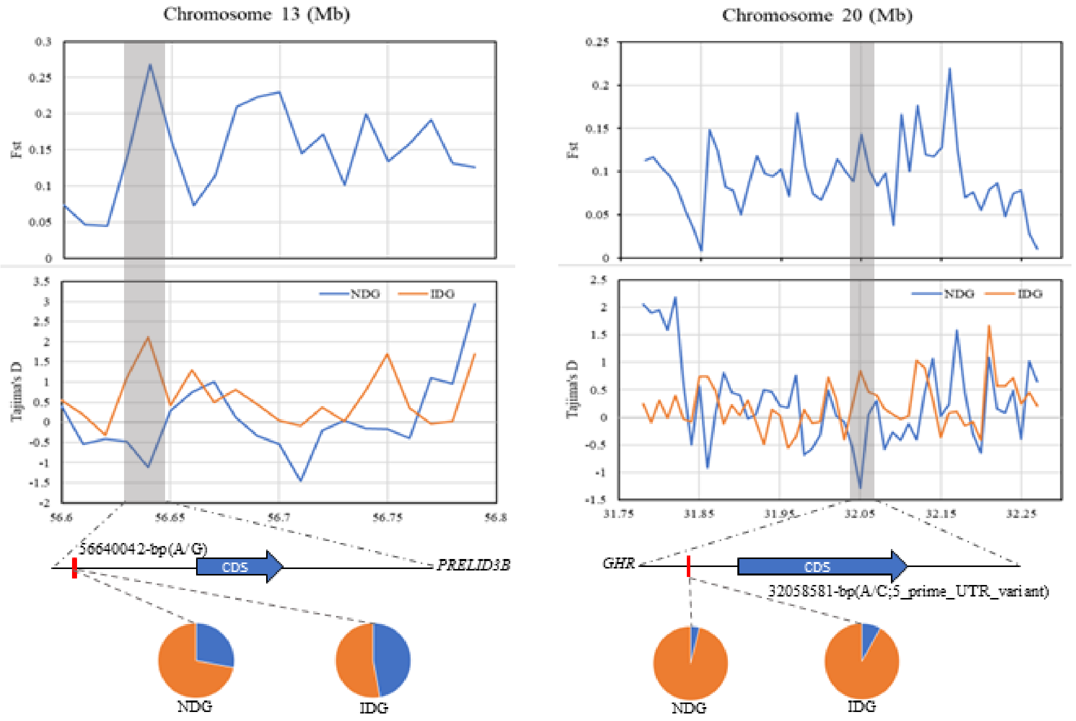

Figure S7 FST value and Tajima's D values around the *PRELID3B* and *GHR* locus.

1.2 Supplemental Tables

Table S1

The data statistic for re-sequenced dairy goat samples.

Population	Abbreviation	Clean_ reads	mapped_ reads	Alignment Rate (%)	Read Depth	Genome Coverage(%)
CRR576035	AAG	525,869,015	513,598,491	0.9767	30.3642	0.9977
CRR576037	AAG	465,472,196	454,602,016	0.9766	26.8912	0.9978
CRR576047	ASG	523,974,951	511,892,588	0.9769	30.2149	0.9981
CRR576048	ASG	516,341,818	504,871,702	0.9778	29.8166	0.9981
CRR576049	ASG	527,057,765	515,303,707	0.9777	30.4151	0.9979
CRR576050	ASG	506,044,564	487,904,644	0.9642	28.6785	0.9981
CRR576051	ASG	487,277,078	470,326,797	0.9652	27.6446	0.998
CRR576052	ASG	512,510,156	494,352,953	0.9646	29.1079	0.9982
CRR576053	GZG	510,276,766	498,861,086	0.9776	29.4967	0.9978
CRR576054	GZG	547,707,371	534,970,518	0.9767	31.6161	0.998
CRR576055	GZG	488,615,611	477,443,623	0.9771	28.2243	0.9981
CRR576056	GZG	547,958,342	527,835,463	0.9633	31.0373	0.998
CRR576057	GZG	561,270,361	540,915,961	0.9637	31.8151	0.998
CRR576058	GZG	474,876,994	458,613,278	0.9658	26.9542	0.9977
CRR576041	NSG	486,744,871	475,495,318	0.9769	28.079	0.9982
CRR576042	NSG	510,428,846	498,887,847	0.9774	29.4588	0.9978
CRR576043	NSG	480,770,104	470,221,528	0.9781	27.7572	0.9982
CRR576044	NSG	479,896,701	462,931,187	0.9646	27.2519	0.9982
CRR576045	NSG	560,942,050	540,761,678	0.964	31.8137	0.998
CRR576046	NSG	559,101,551	538,347,661	0.9629	31.6966	0.998
SRR20216601	GZG	149971502	144713237	0.9649	8.4946	0.996
SRR20216602	GZG	147556995	142258523	0.9641	8.3442	0.9962
SRR20216603	GZG	152485411	147078694	0.9645	8.6242	0.9965
SRR20216604	GZG	167257725	161244297	0.964	9.4664	0.9969
SRR20216605	GZG	151756651	146350734	0.9644	8.5919	0.9966
SRR20216606	GZG	150564958	145130682	0.9639	8.5309	0.9965
SRR20216607	GZG	147726041	142289493	0.9632	8.3615	0.9964
SRR20216608	GZG	154899058	149247926	0.9635	8.7718	0.9966
SRR20216609	GZG	148837733	143445658	0.9638	8.4228	0.9964
SRR20216610	GZG	140940383	135709274	0.9629	7.9532	0.9956
SRR20216611	GZG	149167983	143790909	0.964	8.4446	0.9961
SRR20216612	GZG	145956548	140646018	0.9636	8.2454	0.9959
SRR20216613	GZG	153381771	147842724	0.9639	8.6948	0.9963
SRR20216614	GZG	150287219	144838186	0.9637	8.5013	0.9961
SRR20216615	GZG	146097934	140922620	0.9646	8.2729	0.9963

SRR20216616	GZG	151898589	146450866	0.9641	8.5867	0.9964
SRR20216617	GZG	142454954	137171355	0.9629	8.0314	0.9958
SRR20216618	GZG	147818102	142406651	0.9634	8.3613	0.9964
SRR20216619	GZG	147051633	141694360	0.9636	8.3053	0.9965
SRR20216620	GZG	148669439	143378445	0.9644	8.417	0.9963
CRR281602	LSG	106,846,869	103,077,499	96.47	5.0327	0.9654
CRR281603	LSG	109,789,126	105,988,190	96.54	5.1785	0.9653
CRR281604	LSG	128,084,101	123,788,646	96.65	6.044	0.9809
CRR281605	LSG	119,128,382	114,929,306	96.48	5.6058	0.9706
CRR281606	LSG	99,772,956	96,242,093	96.46	4.6988	0.9575
CRR281607	LSG	103,236,723	99,700,480	96.57	4.8592	0.9655
CRR281608	LSG	108,622,307	104,912,606	96.58	5.1072	0.9729
SRR3565902	LSG	380,621,540	366,843,886	0.9638	20.5117	0.9977
SRR3565904	LSG	387,993,591	372,401,090	0.9598	19.8577	0.9977
CRR576023	NBY	565,393,705	545,369,327	0.9646	32.1512	0.9982
CRR576024	NBY	595,385,965	574,648,731	0.9652	33.8929	0.9984
CRR576025	NBY	656,905,277	633,286,747	0.964	37.3185	0.9983
CRR576026	NBY	473,001,298	462,796,025	0.9784	27.4267	0.9981
CRR576027	NBY	571,545,394	558,658,375	0.9775	33.0363	0.9981
CRR576028	NBY	522,347,300	510,498,403	0.9773	30.1376	0.9983
SRR8224116	NBY	171,500,694	165,673,308	0.966	9.3991	0.992
SRR8224117	NBY	188,059,757	182,478,905	0.9703	10.2482	0.9929
SRR8224118	NBY	223,696,760	217,924,557	0.9742	12.6334	0.9947
SRR8224119	NBY	180,078,754	174,404,774	0.9685	10.1287	0.9931
SRR8224120	NBY	181,414,371	176,133,201	0.9709	9.7392	0.992
SRR8224126	NBY	130,076,651	125,956,220	0.9683	7.3271	0.9881
SRR8224128	NBY	128,977,068	124,832,127	0.9679	7.2675	0.9878
SRR8224129	NBY	161,908,923	157,077,643	0.9702	9.169	0.9909
SRR8224130	NBY	163,144,554	158,371,903	0.9707	9.2313	0.991
CRR281592	TGB	136,923,171	132,455,798	0.9674	6.473	0.9881
CRR281593	TGB	125,072,575	120,983,161	0.9673	5.9091	0.9851
CRR281594	TGB	131,392,296	126,961,668	0.9663	6.2034	0.984
CRR281595	TGB	91,567,896	88,465,849	0.9661	4.3069	0.964
CRR281596	TGB	116,672,655	112,838,156	0.9671	5.5003	0.9812
CRR281597	TGB	130,780,447	126,468,514	0.967	6.2036	0.9868
CRR281598	TGB	117,061,632	113,230,064	0.9673	5.5402	0.9817
CRR281599	TGB	118,583,067	114,543,070	0.9659	5.5691	0.9804
CRR281600	TGB	118,948,165	116,598,726	0.9802	5.7124	0.984
CRR281601	TGB	110,743,548	108,576,414	0.9804	5.3379	0.9807
CRR576029	TGB	538,124,342	525,673,642	0.9769	31.1052	0.9981
CRR576030	TGB	506,870,264	495,230,840	0.977	29.3214	0.9982
CRR576031	TGB	550,554,866	538,154,101	0.9775	31.8606	0.9982
CRR576032	TGB	511,223,068	493,059,008	0.9645	29.0086	0.9981

CRR576033	TGB	518,564,550	500,149,365	0.9645	29.476	0.9981
CRR576034	TGB	473,413,349	456,600,647	0.9645	26.8962	0.9981
CRR576036	NAG	475,376,040	464,622,233	0.9774	27.5088	0.998
CRR576038	NAG	620,503,247	597,140,300	0.9623	35.1203	0.9982
CRR576039	NAG	479,909,352	463,055,601	0.9649	27.2053	0.9979
CRR576040	NAG	564,346,111	544,143,173	0.9642	32.0236	0.998
XN1A	XNG	303,577,603	298,106,545	98.2	17.667	99.73
XN2A	XNG	315,611,671	309,848,547	98.17	18.3726	99.71
XN3A	XNG	425,776,484	417,847,624	98.14	24.8065	99.77
XN4A	XNG	335,476,230	329,105,276	98.1	19.5341	99.73
XN5A	XNG	414,835,251	402,145,087	96.94	23.75	99.75
XN6A	XNG	318,733,255	309,291,076	97.04	18.254	99.74
XN7A	XNG	382,085,482	370,566,988	96.99	21.8894	99.75
XN8A	XNG	319,962,548	310,379,075	97	18.3174	99.75
XN9A	XNG	349,780,816	340,151,467	97.25	20.0117	99.74
XN10A	XNG	317,547,654	308,006,822	97	18.1878	99.74
XN11A	XNG	222,384,209	218,336,240	98.18	12.9504	99.65
XN12A	XNG	236,722,032	229,671,907	97.02	13.5432	99.68
XN13A	XNG	281,375,199	276,363,897	98.22	16.3185	99.71
XN14A	XNG	212,824,183	208,964,328	98.19	12.4428	99.58
XN15A	XNG	305,531,898	300,093,871	98.22	17.7514	99.7
XN16A	XNG	178,167,643	172,835,481	97.01	10.2455	99.49
XN17A	XNG	214,954,416	208,558,087	97.02	12.3117	99.64
XN18A	XNG	213,821,630	207,716,166	97.14	12.2872	99.57
XN19A	XNG	250,254,159	242,765,727	97.01	14.2815	99.68
XN20A	XNG	221,661,860	217,665,123	98.2	12.957	99.63
XN21A	XNG	270,566,003	265,630,272	98.18	15.8103	99.65
XN22A	XNG	236,074,625	231,706,581	98.15	13.8086	99.65
XN23A	XNG	254,124,086	249,549,369	98.2	14.8809	99.66
XN24A	XNG	276,633,019	271,506,354	98.15	16.0537	99.69
XN25A	XNG	215,774,284	211,791,787	98.15	12.4972	99.64
XN26A	XNG	274,705,680	269,736,071	98.19	16.0731	99.66
XN27A	XNG	245,288,708	237,975,495	97.02	14.1154	99.66
XN28A	XNG	231,007,058	224,121,396	97.02	13.3012	99.65
XN29A	XNG	240,187,104	233,022,308	97.02	13.8407	99.64
XN30A	XNG	267,323,955	259,416,824	97.04	15.4024	99.67
XN31A	XNG	178,730,018	173,361,558	97	10.2722	99.54
XN32A	XNG	273,441,962	265,087,005	96.94	15.7221	99.69
XN33A	XNG	277,740,841	269,444,216	97.01	15.9899	99.68
XN34A	XNG	260,579,472	255,102,106	97.9	15.0188	99.67
XN35A	XNG	253,943,424	246,345,795	97.01	14.6114	99.66
XN36A	XNG	325,727,990	316,102,436	97.04	18.7879	99.68
XN37A	XNG	258,170,398	250,493,599	97.03	14.8495	99.67

XN38A	XNG	200,368,909	194,863,341	97.25	11.5301	99.57
XN39A	XNG	226,795,880	220,128,616	97.06	12.988	99.66
XN40A	XNG	187,772,155	182,259,933	97.06	10.8095	99.53
GZ1A	GZG	395,073,410	387,664,244	98.12	23	99.75
GZ2A	GZG	336,756,162	330,511,437	98.15	19.5951	99.76
GZ3A	GZG	422,373,922	414,475,462	98.13	24.4974	99.77
GZ4A	GZG	339,839,562	333,558,093	98.15	19.7983	99.77
GZ5A	GZG	411,641,052	403,904,716	98.12	23.9935	99.76
GZ6A	GZG	456,513,224	442,811,792	97	26.182	99.78
GZ7A	GZG	507,076,914	491,795,551	96.99	29.0559	99.76
GZ8A	GZG	294,979,891	286,051,943	96.97	16.8422	99.7
GZ9A	GZG	419,541,059	406,856,872	96.98	24.0528	99.74
GZ10A	GZG	374,890,538	363,387,848	96.93	21.3794	99.77

Table S2

Statistical analysis of SNP distribution in re-sequenced dairy goat samples.

Type (alphabetical order)	Count	Percent
3_prime_UTR_variant	419,201	0.71%
5_prime_UTR_premature_start_codon_gain_variant	15,559	0.03%
5_prime_UTR_variant	97,910	0.17%
downstream_gene_variant	2,750,237	4.62%
initiator_codon_variant	1	0%
intergenic_region	19,987,360	33.60%
intragenic_variant	1,249	0.00%
intron_variant	32,753,784	55.07%
missense_variant	227,235	0.38%
non_coding_transcript_exon_variant	130,483	0.22%
non_coding_transcript_variant	120	0%
splice_acceptor_variant	1,004	0.00%
splice_donor_variant	1,203	0.00%
splice_region_variant	60,273	0.10%
start_lost	12	0%
stop_gained	17,559	0.03%
stop_lost	17,204	0.03%
stop_retained_variant	9,412	0.02%
synonymous_variant	283,685	0.48%
upstream_gene_variant	2,706,754	4.55%
Transitions	1,010,961,554	
Transversions	431,187,800	

Ts/Tv ratio	2.3446	
